# Menstrual knowledge, sociocultural restrictions, and barriers to menstrual hygiene management in Ghana: Evidence from a multi-method survey among adolescent schoolgirls and schoolboys

**DOI:** 10.1371/journal.pone.0241106

**Published:** 2020-10-22

**Authors:** Shamsudeen Mohammed, Roderick Emil Larsen-Reindorf

**Affiliations:** 1 Department of Population, Family & Reproductive Health, School of Public Health, Kwame Nkrumah University of Science and Technology, Kumasi, Ghana; 2 College of Nursing & Midwifery, Population and Reproductive Health Resident, Nalerigu, Ghana; 3 Department of Obstetrics & Gynaecology, School of Medical Science, Kwame Nkrumah University of Science and Technology, Kumasi, Ghana; Helen Keller International, SIERRA LEONE

## Abstract

On a daily basis, schoolgirls in low and middle-income countries discover blood on their clothing for the first time in school environments without toilets, water, or a supportive teacher, mentor, or role model to help them understand the changes happening in their bodies. This study aimed to examine the menstrual knowledge, sociocultural restrictions, and barriers to menstrual hygiene management in school environment among adolescent schoolgirls in a rural community. We collected quantitative data from 250 adolescent schoolgirls and qualitative data from thirty schoolboys and five schoolteachers in five Junior High Schools in the Kumbungu district of northern Ghana. Binary logistic regression models were fitted to determine the predictors of poor menstrual knowledge. Qualitative data were transcribed verbatim, coded, and organized into themes. Overall, 53.6% of the girls had poor knowledge about menstruation. Most of the boys had heard about menstruation and had an idea about what menstruation is with most of them describing it as “the flow of blood through the vagina of a female.” The boys revealed that terms such as “*Vodafone*,” “*Red card*,” and “*Palm oil*” are used to describe menstruation in the schools and within the community. After adjusting for the effect of other sociodemographic factors, we found evidence that girls in their late adolescents were less likely to have poor menstrual knowledge compared to those aged 10–14 years (aOR 0.20, 95%CI 0.08–0.48). Maternal education was protective against poor menstrual knowledge. When compared to adolescents whose mothers were illiterates, those whose mothers had basic education (aOR 0.62, 95%CI 0.28–1.40) and those whose mothers had secondary or higher education (AOR 0.22, 95%CI 0.06–0.76) were less likely to have poor knowledge about menstruation. Adolescents from homes with no television and radio sets were more likely to have poor menstrual knowledge compared to those from homes with television and radio sets (aOR 2.42, 95%CI 1.41–4.15). Comfort, safety, and cost were the major factors that influenced their choice of sanitary products. Most of the teachers said the schools do not provide students with sanitary products, even in emergencies. We found that girls were not to prepare some local dishes (*e*.*g*. *Wasawasa*) during their periods and are forbidden from participating in religious activities (i.e. read the Holy Quran or pray in the mosque) during the period of menstruation. Open discussions about menstruation and its management are not encouraged and girls are considered unclean and impure during the period of menstruation. None of the schools had a regular supply of water in WASH facilities, a mirror for girls to check their uniforms for bloodstains or soap in the toilet facilities for handwashing. Menstrual education through the standard school curriculum, starting from primary school, could prepare girls for menarche, improve their knowledge on menstruation, and teach boys how to support girls and women during the period of menstruation. This could also eliminate the sociocultural misconceptions surrounding menstruation.

## Introduction

On a daily basis, schoolgirls in low- and middle-income countries discover blood on their clothing for the first time in settings without toilets, water, or a supportive teacher, mentor, or role model to help them understand the changes happening in their bodies [1]. A girl’s first experience of seeing blood (menarche) from her vagina can be frightening and shocking [2, 3]. Yet, the physiological basis of menstruation, biological changes at puberty, the menstrual cycle, infection risks posed by poor practices, and the material disposal options available to girls are hardly ever discussed openly. The silence surrounding it burdens young girls by keeping them ignorant of this natural phenomenon [4–7]. Even when adolescents seek information about menstruation, adults usually feel shy, uncomfortable, and reluctant to discuss it because of socio-cultural and religious misconceptions and proscriptions. Due to this, girls are not properly educated about what is happening to their bodies and how to stay healthy and maintain their self-esteem during menstruation [2, 6, 8]. When mothers decide to provide information, it is usually on how to practice rituals and restrictions during menstruation [2, 3, 7, 9]. In most cases, adult women themselves are unaware of the biological facts of menstruation or the good hygienic practices required, instead, they pass on cultural taboos and restrictions to be observed [2, 3, 7, 9].

Furthermore, many low- and middle-income countries have very limited education in schools about menstruation. Classroom teachers may be unwilling to discuss menstrual hygiene management, particularly male teachers, due to the taboos associated with menstruation in these settings [10]. In some situations, the teachers themselves are uninformed [10]. Consequentially, adolescent girls have no option than to seek information outside of the formal learning environment [3, 8, 10]. In a study among adolescent girls in Nigeria, it was reported that only 33.8% of the girls knew that the range of a single menstrual cycle is from day one of menstruation to the beginning of the next menstruation and only 2.5% of the girls were aware that a normal menstrual cycle varies between 21 to 35 days [11]. Although 56.5% of the adolescents in the study knew a woman could become pregnant when she engages in unprotected sex during a certain phase of her menstrual cycle, none of them knew that phase of the menstrual cycle [11]. In India, more than 75% of girls surveyed did not know the source of the menstrual blood [12].

The period of adolescence is of particular concern because, in low- and middle-income settings, myths, taboos, and socio-cultural restrictions create barriers for adolescents to acquire accurate information about menstruation. This limits their daily and routine activities, and have the potential to negatively affect their self-esteem, reproductive health, and schooling [2, 9, 13]. For instances, in some cultures, it is taboo for women and girls to bath during their periods, touch a cow, or look in a mirror; they are excluded from water sources, food that others will eat or cooking activities, religious rituals, sanitation amenities and the family home [2, 9, 12, 14–16]. Studies in India found that adolescents are restricted from attending school during their menstrual period or from wearing new clothes in some communities in Indian [12, 16]. In a comparative study among girls in Ghana, Ethiopia, Cambodia, and Tanzania, it was discovered that girls from Tanzania and Ghana were told improper disposal of used sanitary materials would lead to infertility, while the majority of the girls from Cambodia were instructed by mothers to keep their first used menstrual sanitary material, as it was believed to offer protection from others bad intentions, serve as anti-snake venom for snakebite, and promote smooth skin [17]. In Ethiopia bathing during menstruation was believed to lead to heavier menstrual bleeding [17]. Formerly, menstruating women and girls in western Uganda were prohibited from drinking milk because it was believed to affect milk production from cows [5]. Similarly, women and girls in eastern Uganda were forbidden from seeding groundnuts during their periods because it was thought to affect yield while in central Uganda menstruation was regarded as a secret only known to oneself [5].

At school, adolescent girls in low-income countries are faced with poor hygiene facilities such inadequate water for washing, lack of soap, poor privacy, non-functioning or unclean toilets and no disposal facilities to support the hygienic management of menstruation in the school environment [1, 4, 18]. The poor and inadequate sanitation situation in the schools may prevent girls from attending school, particularly during their periods [1, 4, 19]. Findings of a study in Ethiopia revealed that over 80% of students do not change their menstrual absorbent materials in school; they prefer to do so at home mainly because of lack of suitable water, sanitation, and hygiene facilities in the schools [20]. Also, studies have demonstrated a link between inadequate water, sanitation, hygiene facilities, and school absenteeism among adolescent girls in low-income settings [9, 21]. Furthermore, to stay healthy and avoid physical discomfort and leakages, adolescent girls need information on the types of menstrual absorbent materials and how to use, wash, and dispose of them hygienically [18]. Findings of a study done among schoolgirls in Ethiopia showed that 35.4% of the students used sanitary napkins, 55.6% of them used homemade cloth, and 9% used underwear as absorbent materials for menstrual blood [20].

Men and adolescent boys have an important role to play in supporting girls in their menstrual hygiene management, as brothers, peers or colleagues [2]. Men and boys can be activated to break the barriers and taboos around menstruation, spend money on menstrual products or a toilet to increase the menstrual health of women and girls and provide a good example to their sons by supporting menstrual health [6]. However, the perspectives and knowledge of men and boys on menstruation and menstrual hygiene management are inadequately research and therefore poorly understood [22], in particular, their perspectives on the challenges adolescents face in a school environment during their menstrual periods. A review of 44 studies on menstrual knowledge among adolescents in low- and middle-income countries found that boys were rarely included in the studies reviewed [22].

The study aimed to assess the barriers to menstrual hygiene management among adolescent schoolgirls in a rural community in northern Ghana. The specific objectives of the study were 1) to assess the menstrual knowledge of the adolescent schoolgirls 2) to examine adolescents access to menstrual hygiene management products in a rural community, 3) to explore the socio-cultural and religious restrictions on adolescents during menstruation, 4) to assess the availability and suitability of water, sanitation, and hygiene facilities for the management of menstruation in a school environment, and 5) assess the menstrual knowledge of adolescent schoolboys and the perspectives of teachers on menstrual hygiene management in a school environment. The findings are expected to improve policies, campaigns, and programmes on menstrual education to include men and boys. Such inclusion will provide boys with a balanced and accurate knowledge base and therefore help towards reducing the social stigma around menstruation that is often experienced by young girls [23].

## Materials and methods

This cross-sectional study was conducted in five Junior High Schools (JHS) in Kumbungu in the Northern Region of Ghana as part of a larger study to measure menstrual hygiene management and its impact on school absenteeism [19]. All the residents in the Kumbungu district live in rural communities with the population equally distributed between males and females [24]. Dagombas are the indigenous group of people in Kumbungu and the predominant religion in the area is Islam. Most households in the district do not have toilets and the most widely used method of solid waste disposal is by the public dump in the open space [24]. We collected quantitative data from adolescent schoolgirls aged 10 to 19 years old and qualitative data from schoolboys and schoolteachers in all the five Junior High Schools in Kumbungu. Adolescents who had not attained menarche and those outside the five Junior High Schools were excluded.

### Sample size and sampling

**Fig 1:**
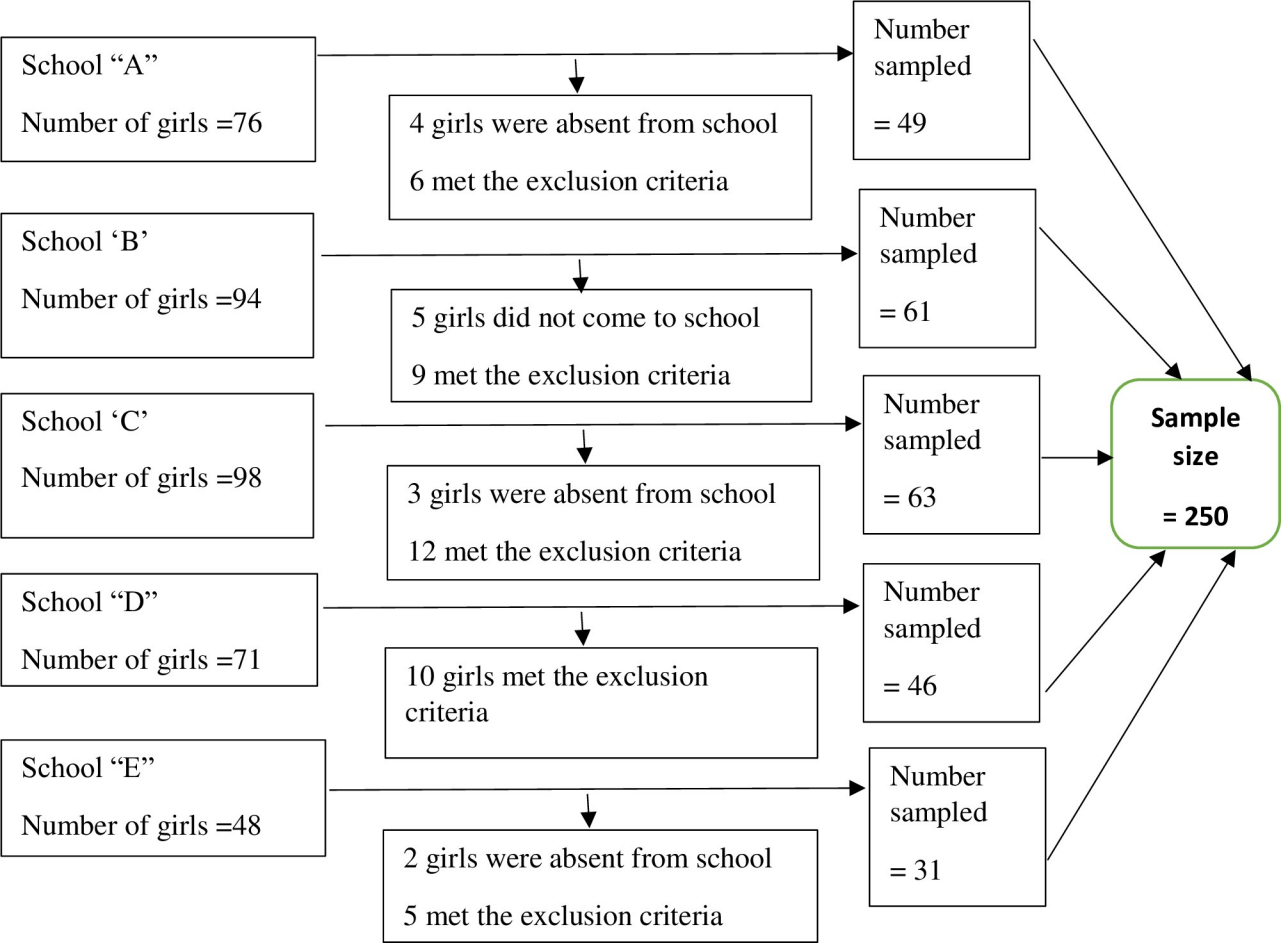
Proportional sampling of participants.

A sample size of 250 was estimated using Cochran’s correction formula for finite population [25] based on 57% prevalence of menstrual knowledge from a previous study in Ghana [26], 95% confidence interval, 5% level of precision, 30% non-response rate, and a population of 387 adolescent school girls in the five Junior High Schools. Upon approval from the District Education Director and permission from the school heads, the computed sample size of 250 was proportionally allocated to each of the five schools. The proportional allocation was computed using the formula, $n_{i}=n\frac{N_{i}}{N}$, where n represents sample size, Ni represents the number of adolescents in each of the schools and N represents the total number of adolescents in all the five schools. The proportion to size allocation of the sample size is presented in Fig 1. The research team then generated a table of random numbers with OpenEpi version 3, which was compared to a sampling frame to sample participants for the study in each of the five schools using simple random sampling method. This sampling method gave each adolescent girl between the ages of 10 to 19 years an equal chance of being selected for the study. For the qualitative aspect, purposive and convenient sampling methods were used to select 30 adolescents from three of the five Junior High schools for focused group discussions (FGDs) and a teacher from each of the five schools for key informant interviews (KIIs). Three schools were selected for the FGDs because the students of other schools were engaged in an examination during the period the FGDs were conducted.

### Data collection

A pretested self-administered questionnaire was used to collect quantitative data on adolescents menstrual knowledge, access to menstrual hygiene materials, sociocultural and religious restrictions on menstruation, and the availability of suitable water, sanitation, and hygiene facilities in the schools (see S1 Questionnaire). Data on the age of the adolescents, age at menarche, religious affiliation, and the educational level of their parents were collected. The data collection instruments were designed after a thorough review of relevant literature and previous studies on menstruation and barriers to menstrual hygiene management [4, 12, 20, 27–30]. The questionnaire was piloted among 30 adolescents with similar characteristics as the study population in a similar Ghanaian setting. The purpose of the pre-test was to ascertain the clarity, sensitivity, and practicability of the questionnaire and to identify ambiguous and poorly constructed items as well as other problems that may be encountered during data collection.

We recruited and trained three data collectors to acquaint them with the objective and purpose of the study; how to obtain consent, ensure confidentiality of the information and on the rights of the participant during the study. Because of the culturally sensitive nature of menstruation and menstrual hygiene management in rural communities, only female data collectors were recruited for the data collection involving adolescent girls. The data collectors distributed the questionnaires to the girls in classrooms with the help of schoolteachers. The girls were positioned apart from each other on their desk and the study questionnaire explained to them by the data collectors. They were encouraged not to consult their colleagues in responding to the questionnaire and asked to contact the data collectors if they need assistance. The girls were allowed enough time to complete the questionnaire and all completed questionnaires were presented to the data collectors who moderated the data collection process in the classrooms. The first author reviewed the questionnaires on daily basis to ensure completeness and consistency. In addition, regular meetings were held with data collectors to identify and address challenges faced during data collection. After manually checking data for completeness, questionnaires were numbered to avoid double entry.

For the qualitative data collection, trained research assistants used a discussion and an interview guide to moderate the FGDs and KIIs, respectively. The FGD and KII guides contained questions on the menstrual knowledge of adolescent schoolboys and the perspectives of both teachers and adolescent schoolboys on the menstrual hygiene management of girls in a school environment and the challenges they face. In the three schools were FGDs were held, the boys sat in a circle on their desk with a male moderator in an empty classroom with doors and windows shut to provide privacy. Questions were posed by the moderator and probes used occasionally to solicit more response when necessary. The boys were assigned numbers to ensure anonymity and all the discussions were recorded with a tape recorder. Overall, 30 boys were engaged in the FGD, 10 participants in each of the three separate discussions in the three schools selected for FGD. Interviews with teachers were conducted in offices with two data collectors, a male and a female. The interviews were recorded with a tape recorder and notes taken to capture the complete thoughts and perspectives of the teachers. A total of five teachers were interviewed, one teacher from each school. The appropriateness of water, sanitation, and hygiene (WASH) facilities in the schools for the management of menstruation was assessed using a checklist developed based on UNICEF guidelines for menstrual appropriate WASH facilities [29].

### Data management and analysis

Quantitative data were analysed with Stata v14.0 and presented in tables as percentages, frequencies, proportions, means, and standard deviations. The adolescents overall knowledge about menstruation was measured out of seven knowledge specific questions (Table 2). Each correct response earned one point while an incorrect response earned no point. The total scores of the participants were calculated and used to determine their knowledge of menstruation. Participants were classified as having good knowledge of menstruation if they scored 5–7 points and poor knowledge of menstruation if they scored 0–4 points. Binary logistic regression was then used to determine the sociodemographic factors associated with the adolescent’s menstrual knowledge. Univariable analysis was initially performed and variables that showed strong or statistically significant (p < .05) effect were considered for multivariable analysis. We adjusted for the effect of other sociodemographic factors in the multivariable analysis to determine the independent predictors of adolescents menstrual knowledge. Odds ratios, 95% confidence interval, and likelihood ratio p values were presented for all the factors and variables were only retained in the final multivariable regression model if they showed a likelihood p-value of <0.05. Data (audio recordings) from FGDs and KIIs were transcribed verbatim and analysed using thematic data analysis. A printout of the transcript was read several times and the sections relevant to the study objectives coded using open coding. The codes were discussed and modified where necessary. We then organised the codes into broader themes based on the patterns of the codes. The themes were defined, and descriptive narrations written and used to support the quantitative data with quotes from the participants.

### Ethics statement

Ethical approval for the study was sought from the Human Research and Publication Ethics Committee of the Kwame Nkrumah University of Science and Technology (CHRPE/AP/406/17). All the participants were informed of the purpose of the study, their right to decline to answer any question or withdraw from the study at any time without any condition or threat. In addition, a participant information sheet containing detailed information about the rights of the participants, the purpose, and significance of the study was attached to the questionnaire. Each participant indicated consent for participation by signing a consent form designed for the study. For participants under the age of consent, informed verbal consent was obtained from headteachers of their respective schools and signed informed assent from the participants. Parental consent was not obtained since headteachers act as guardians in School. The consent procedure was approved by the ethics committee and accepted by the headteachers. Confidentiality of the information collected was ensured.

## Results

### Sociodemographic characteristics of the participants

Table 1 presents the sociodemographic characteristics of the participants. Most of the girls were 15–19 years old (85.6%), only 36 (14.4%) of them were between the ages of 10–14 years. The mean age at menarche was 13.21 (SD = 1.54) years. More than half of the girls aged 10–14 years (63.9%) and those aged 15–19 years (51.9%) had poor menstrual knowledge. Those who experienced menarche during late adolescents (14–17 years) had better menstrual knowledge than girls who experienced menarche between age 10–13 years at the time of the study. One hundred and seventy-three (69.2%) of the participant's father and 204 (81.6%) of their mothers were illiterates. A higher proportion (75.0%) of adolescents whose mothers attained secondary or higher education had appropriate knowledge about menstruation, while more than half (56.9%) of those whose mothers had no formal education demonstrated poor knowledge about menstruation. Nearly 55% of the girls who indicated they had television or radio in their homes had good menstrual knowledge.

**Table 1:** Sociodemographic characteristics of adolescent schoolgirls.

Characteristics	Knowledge about menstruation		Total (%)
	Good (%)	Poor (%)	
**Age group (years)**			
10–14	13(36.1)	23(63.9)	36(14.4)
15–19	103(48.1)	111(51.9)	214(85.6)
Mean (SD)			15.9(1.64)
**Age at Menarche**			
10–13	56(42.8)	75(57.3)	131(52.4)
14–17	60(50.4)	59(49.6)	119(47.6)
Mean (SD)	13.2(1.54)		
**Religious affiliation**			
Christianity	5(83.3)	1(16.7)	6(2.4)
Islam	111(45.5)	133(54.5)	244(97.6)
**TV/Radio**			
Yes	72(54.6)	60(45.5)	132(52.8)
No	44(37.3)	74(62.7)	118(47.2)
**Father's Education**			
No education	76(43.9)	97(56.1)	173(69.2)
Basic Education	21(46.7)	24(53.3)	45(18.0)
Secondary and above	19(59.4)	13(40.6)	32(12.8)
**Mother's Education**			
No education	88(43.1)	116(56.9)	204(81.6)
Basic education	16(53.3)	14(46.7)	30(12.0)
Secondary and higher	12(75.0)	4(25.0)	16(6.4)
**Father's Occupation**			
Trader	9(56.3)	7(43.8)	16(6.4)
Farmer	74(46.8)	84(53.2)	158(63.2)
Formal sector employee	4(26.7)	11(73.3)	15(6.0)
Unemployed	18(47.8)	20(52.6)	38(15.2)
Others	11(47.8)	12(52.2)	23(9.2)
**Mother's Occupation**			
Trader	24(41.4)	34(58.6)	58(23.2)
Farmer	35(50.0)	35(50.0)	70(28.0)
Formal sector employee	8(38.1)	13(61.9)	21(8.4)
Unemployed	41(51.9)	38(48.1)	79(31.6)
Others	8(36.4)	14(63.6)	22(8.8)

### Menstrual knowledge of adolescent schoolgirls

**Fig 2:**
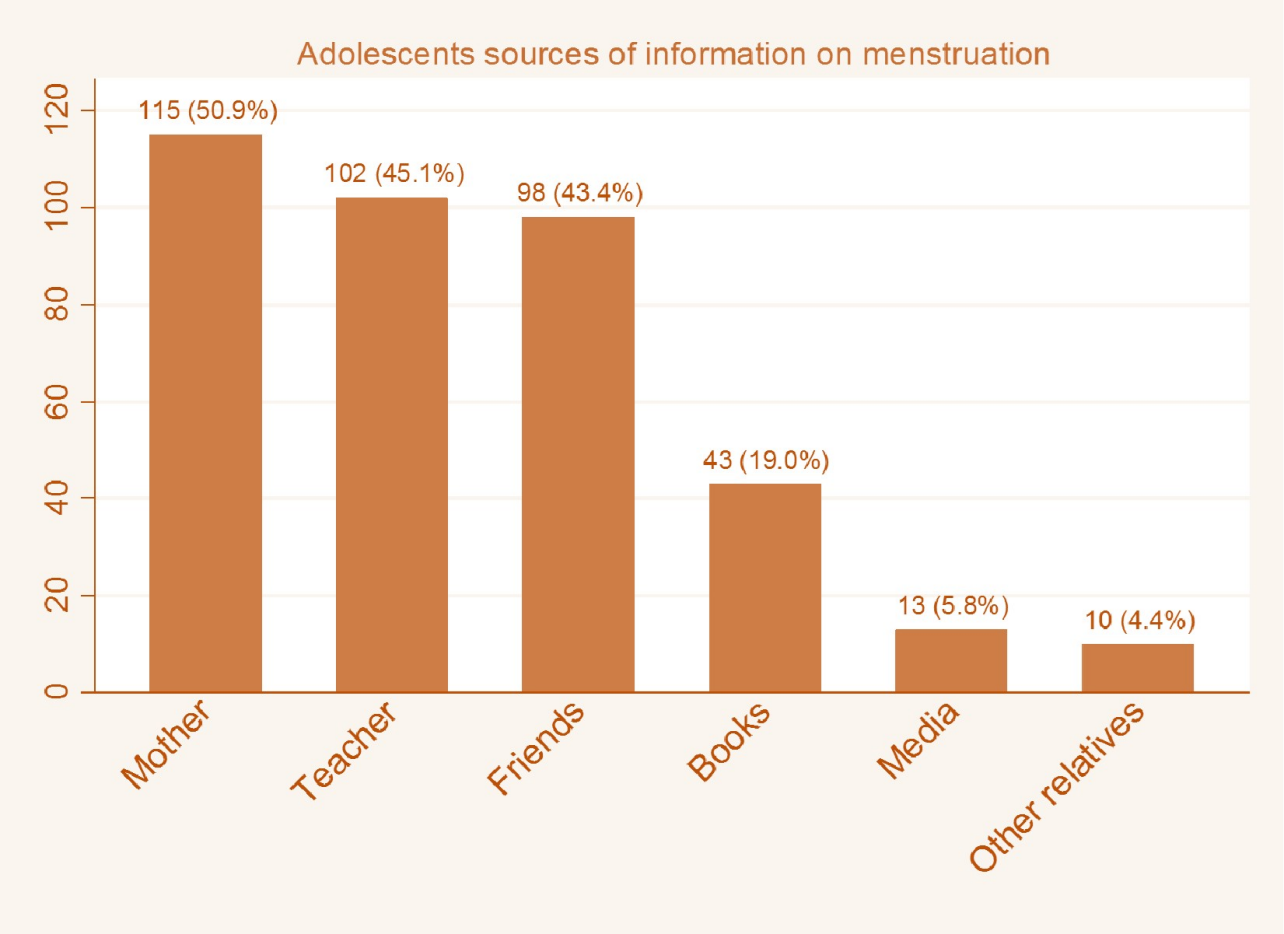
Adolescents sources of information about menstruation.

Participants knowledge about menstruation is presented in Table 2. The results show that 81.6% of the girls knew menstruation is a normal physiological process, while 86.8% incorrectly believed the vagina is the source of menstrual blood. Only 2.8% of them knew that the source of menstrual blood is the uterus. Most of the participants said the normal age of menarche is 10–14 years (69.2%), the normal number of days for menstrual bleeding is 5–7 days (67.6%), and that the length of the menstrual cycle is 21–35 days (68.8%). Many of the girls had heard about menstruation before their first menstrual period. As shown in Fig 2, of the two hundred and nineteen who knew about menstruation before menarche, 50.9% got information from their mothers, 45.1% from teachers, 43.4% from friends, 19.0% from books, 5.8% from media, and 4.4% from other relatives. One hundred and eight (72%) participants believed sugary foods should not be consumed during menstruation; only 7.6% of the girls knew that there is no food restriction during menstruation. Results of the overall knowledge score show that 46.4% of the girls in this study had good knowledge about menstruation while 53.6% demonstrated poor knowledge about menstruation.

**Table 2:** Adolescent schoolgirls knowledge about menstruation.

Variables	Number	Percent
**Menstruation is**		
Normal Physiological process	204	81.6
Not a Normal Physiological process	46	18.4
**Source of menstrual blood**		
Abdomen	13	5.2
Bladder	13	5.2
Vagina	217	86.8
Uterus	7	2.8
**Perceived normal age at menarche**		
10–14 years	173	69.2
15–18 years	77	30.8
**Duration menstrual bleeding**		
3–4 days	72	28.8
5–7 days	169	67.6
8–9 days	9	3.6
**Duration of menstrual cycle**		
< 21 days	67	26.8
21–35 days	172	68.8
> 35 days	11	4.4
**Heard about menstruation before menarche**		
Yes	219	87.6
No	31	12.4
**Foods to avoid during menstruation**		
Sugary foods	180	72
Oily Foods	15	6
Milk products	28	11.2
No food restriction	19	7.6
Meat	4	1.6
Eggs	4	1.6
**Overall knowledge about menstruation**		
Good Knowledge	116	46.4
Poor Knowledge	134	53.6

### Menstrual knowledge of adolescent schoolboys

Focused group discussions with adolescent boys revealed that most of them had heard about menstruation and had an idea about what menstruation is about, even though some of them revealed that it is scarcely discussed among boys. The boys were able to describe what menstruation is with most of them describing it as “the blood that comes out of a girl’s vagina” and “the flow of blood through the vagina of a female.” They understood the onset of menstruation as a sign of maturity and a girl’s ability to become pregnant. Most of them described it as a natural process. In general, the boys anticipated girls to start menstruating between the ages of 10 to 19 years.

*“Menstruation is the monthly flow or discharge of blood from a girl’s vagina when they reach maturity or at their adolescent stage” (FGD*, *boy)*.

*“Girls menstruate in order to give birth” (FGD*, *boy)*.

The boys indicated that terms such as “Vodafone”, “Red card”, and “palm oil” are used to describe menstruation in the school and the community. They, however, said the terms are mostly used by the girls and females in the school and the community to describe the period during which they menstruate. The boys mentioned they acquired information about menstruation from teachers and friends.

*“We learn about puberty and menstruation in the class from our science teacher” (FGD*, *boy)*.

### Sociodemographic factors associated with menstrual knowledge of adolescents

The association between the sociodemographic characteristics and menstrual knowledge of the adolescents is presented in Table 3. After adjusting for the effect of other sociodemographic predictors, we found evidence that the adolescents age (p<0.001), mothers’ education (p = 0.023), and ownership of television and radio (p = 0.001) were associated with poor menstrual knowledge. Girls in their late adolescents were 80% less likely to have poor menstrual knowledge compared to those aged 10–14 years (aOR 0.20, 95%CI 0.08–0.48). Maternal education was protective against poor menstrual knowledge. When compared to adolescents whose mothers were illiterates, those whose mothers had basic education (aOR 0.62, 95%CI 0.28–1.40) and those whose mothers had secondary or higher education (aOR 0.22, 95%CI 0.06–0.76) were less likely to have poor knowledge about menstruation. Adolescents from homes with no television and radio sets were more likely to have poor menstrual knowledge compared to those from homes with television and radio sets (aOR 2.42, 95%CI 1.41–4.15).

**Table 3:** Association between sociodemographic characteristics and menstrual knowledge of adolescent girls.

Characteristics	Crude odds ratio (95% CI)	p-value	Adjusted odds ratio (95% CI)	p-value
**Age group (years)**				
10–14	1		1	
15–19	0.28(0.12–0.64)	0.001	0.20(0.08–0.48)	<0.001
**Age at Menarche**				
10–13	1			
14–17	0.73(0.45–1.21)	0.224		
**Father's Education**				
No education	1			
Basic Education	0.90(0.46–1.73)	0.273		
Secondary and above	0.54(0.25–1.15)			
**Mother's Education**				
No education	1		1	0.023
Basic education	0.66(0.31–1.43)	0.032	0.62(0.28–1.40)	
Secondary and above	0.25(0.08–0.81)		0.22(0.06–0.76)	
**TV/Radio**				
Yes	1		1	
No	2.02(1.22–3.35)	0.006	2.42(1.41–4.15)	0.001

### Adolescent schoolgirls access to menstrual hygiene materials

Adolescent girls’ access to menstrual absorbent materials is presented in Table 4. Most of the girls often used commercial sanitary pads (60.7%) and reusable cloth pad (54.2%). The results show that the participants choice of absorbent material was influenced by comfort (39.2%), followed by safety (26.8%), cost (4%), and availability (6.8%). Cost of menstrual care products (75.7%) was cited as the major reason for the inability of one hundred and fifteen (46%) of the adolescents to purchase a sanitary pad two months before the study.

**Table 4:** Adolescent schoolgirls access to menstrual absorbent materials (n = 250).

Variables	Number	Percent
**Frequently used Absorbent Material**^a^		
Disposable sanitary pad	150	60.73
Reusable cloth pad	134	54.24
Disposable Cloth	40	16.19
Others	67	27.13
**What influence your choice of absorbent materials**		
Comfort	98	39.20
Safety	67	26.80
Cost	35	14.00
Availability	17	6.80
Ease of disposal	14	5.60
Ease of re-use	19	7.60
**Availability of sanitary pad for sale in shops in your town**		
Yes	215	86.00
No	27	10.80
I don't know	8	3.20
**Bought sanitary pad from a shop in the last two months**		
Yes	135	54.00
No	115	46.00
**Why you have not bought sanitary pad (n = 115)**		
I still have some pads	16	13.91
I can’t afford the cost of sanitary pad	87	75.65
My parents buy for me	7	6.09
Others	5	4.35

^a^ multiple responses were allowed and the percentage is greater than 100.

### Adolescent boys’ perspective on girls’ access to menstrual hygiene materials

When asked about sanitary products, the schoolboys stated that girls use sanitary pad and cloth when they are menstruating to absorb menstrual blood. They further explained that girls use these materials “*to avoid staining their clothes*”, “*not to feel shy when the blood flows out*,” and “*to suppress the scent of the blood from coming out*”. They claimed that sanitary pads were not very expensive and are sometimes provided by a local Non-Governmental Organisation (NGO), CAMFED.

*“They use cloths and pads; they use it to absorb the blood that comes out from the vagina” (FGD*, *boy)*.

*“They can get the pad in stores because it is not costly and some NGO’S do provide them with sanitary pads” (FGD*, *boy)*.

### Schoolteachers’ perspective on girls’ access to menstrual hygiene materials

Of the five teachers that were interviewed, only one teacher said the school provides sanitary pads for girls during menstrual emergencies. The rest of the teachers said their schools do not provide students with sanitary materials, even in emergencies. They, however, mentioned that a local NGO, CAMFED, sometimes provide the girls with sanitary pads. The teachers cited the lack of sanitary pads in schools and the cost of sanitary pads as some of the challenges the schoolgirls face during their periods.

*“The school does not provide sanitary materials to students*, *is only CAMFED that provide the girls with some sanitary materials” (KII*, *teacher)*.

*“It is only in some instances that the teachers buy for those who come to school and have their menses” (KII*, *teacher)*.

### Cultural and religious beliefs about menstruation

Table 5 presents the cultural and religious beliefs about menstruation in the study area. We found that abstinence from religious activities (85.7%) was the most common restriction among the two hundred and fifty respondents followed by the misconception that girls who are menstruating are unclean and impure (73.2%), and the prohibition of discussions about menstruation and its management (36.4%).

**Table 5:** Cultural and religious restrictions on menstruation (n = 250).

Restrictions^a^	Number (%)
Don't discuss menstruation and its management	*87 (36*.*40%)*
Don't perform house chores	*36 (15*.*06%)*
Don't perform religious activities	*205 (85*.*77%)*
Menstruating girls are unclean and impure	*175 (73*.*22%)*
Sleep separately from other family members	*35 (14*.*64%)*

^**a**^ multiple responses were allowed and the percentage is greater than 100.

The discussions with the boys revealed that girls who are menstruating are not to prepare some local dishes (e.g. Wasawasa), read the Holy Quran, pray in the mosque, or attend some ceremonies.

*“They are not supposed to pray in the mosque when they are menstruating” (FGD*, *boy)*.

### Water, sanitation, and hygiene facilities for menstrual hygiene management in the schools

As illustrated in Table 6, all the five (5/5) Junior High Schools in the study area had toilet facilities, separate block of toilet for boys and girls, and the toilet and wash facilities had doors. Two of the five (2/5) schools had clean toilet facilities and functioning handwashing facilities outside the toilets. None (0/5) of the schools had a mirror for girls to check their uniforms for bloodstains or soap in the toilet facilities for handwashing. The schools also lacked (0/5) regular supply of water.

**Table 6:** Water, sanitation, and hygiene facilities for menstrual hygiene management in the Junior High Schools.

Variable	Proportion (n = 5)^a^
Toilet facility in the school	*5/5*
Separate block of toilet for boys and girls	*5/5*
Toilet and wash facilities have doors	*5/5*
Adequate space in toilet	*4/5*
Toilet facility is easily accessible	*4/5*
Toilet facility is clean	*2/5*
Mirror in the toilet	*0/5*
Disposal facilities in the toilet	*1/5*
Functioning hand washing facility	*2/5*
Regular supply of water in the toilet facility	*0/5*
Regular supply of soap in the toilet facility	*0/5*

^a^the denominator denotes the number of schools in which WASH facilities were assessed and the numerator represents the number of schools that had the facility assessed.

### Perspectives of teachers on water, sanitation, and hygiene facilities

The teachers said the schools had separate toilet facilities for both boys and girls. The interviews revealed that most the schools had water facilities (Veronica buckets) outside the toilets and students were expected to carry water into the toilet facilities for use. In one of the schools, a teacher said students fetch water from nearby houses because the school does not have a standpipe. Most of the teachers disclosed that there were no disposal facilities (e.g. dustbins) for the disposal of used menstrual absorbent materials in the toilets. The teachers stated that some of the girls prefer to dispose of used pads in the latrines rather than in dustbins and that, the dustbins are stolen when placed in the toilets. According to some of the teachers, the schools only provide soap for handwashing when they receive money to buy supplies.

*“Well*, *the toilet facility I will say is enough because this is a cluster of schools*, *so every school has a separate toilet for both the girls and the boys” (KII*, *teacher*, *Kumbungu)*.

*“We have a standpipe in the school which usually flow during the raining season and it is on and off during the dry seasons” (KII*, *teacher*, *Kumbungu)*.

*“Almost every day the pipe runs but is just that the school here we don’t have a pipe*, *so we fetch from the primary school and the nearby houses” (KII*, *teacher*, *Kumbungu)*.

*“The school does not have dustbins in the toilets*. *We only have a single dustbin for our (teachers) use*. *The children will tell you that in their homes they are advised not to throw the pads into dustbins*, *they should rather dispose in the latrines and that is what they do here“(KII*, *teacher*, *Kumbungu)*.

*“We don’t provide soap on regular basis; it is once in a while we provide soap” (KII*, *teacher*, *Kumbungu)*.

## Discussion

Accurate information on menstruation and menstrual hygiene management is crucial for women and girls to manage their periods with confidence and dignity and be able to make informed decisions about their menstrual health [31]. In this study, we found that most of the girls had heard about menstruation before their first menstrual period, which is in line with what was reported in previous studies [9, 20, 28]. The adolescent girls main source of information about menstruation was their mothers which is consistent with similar studies among adolescent girls in several low and middle-income countries [2, 3, 7, 9, 32]. However, more than half of the girls in this study had poor knowledge about menstruation which contradicts the findings of previous studies [20, 28]. This means that even though the adolescents were aware of menstruation before menarche, most of them were poorly prepared to experience their first menses. Findings of a scoping review involving 44 studies from 12 low- and middle-income countries support this claim when it revealed that adolescent in resource constrain settings are under-prepared for puberty and menstruation [22].

The poor knowledge of the adolescents may be attributed to insufficient knowledge of their main source (mothers) of information on menstruation as studies in low and middle-income countries have shown that mothers themselves lack sufficient and accurate knowledge on menstruation and the biological process involve and may pass on taboos and restrictions to adolescents [2, 3, 22, 33]. In addition, discussions on menstruation and puberty in most Ghanaian communities and schools are inadequate especially in rural areas due to misconceptions and superstitions [14]. The poor knowledge about menstruation among the adolescents could result in misconceptions and misunderstanding about menstruation [13, 33].

We found evidence that the adolescents age, mothers’ education, and ownership of television and radio were associated with poor menstrual knowledge. Despite the low knowledge score in this study, most of the girls knew menstruation is a normal physiological process; they correctly stated the length of the menstrual cycle, the age of menarche, and the length of menstrual bleeding. This suggests that adolescent girls are not completely ignorant of menstruation, and menstrual education programmes in resource constrain setting should build on this to provide accurate information on the mechanism of menstruation and detail explanation on what happens to the body of a woman or a girl during menstruation.

House *et al*., observed that men and boys have an important role to play in supporting women and girls in their menstrual hygiene management [2], particularly in patriarchal communities such as those in Northern Ghana where men have control over financial resources and often make decisions that affect the health of women and girls. An understanding of menstruation and the challenges associated with it could help them become more understanding and supportive to women and girls. Most of the adolescent boys in this study had heard about menstruation and had an idea about what menstruation is, with most of them describing it as a natural process. However, they had inadequate knowledge about the process of menstruation. Several studies in low and middle-income countries have reported similar findings among adolescent boys [34–36]. This situation underscores the need to expand the education on menstruation and menstrual hygiene management to include men and boys as this could help them develop an appropriate and accurate understanding of the mechanism of menstruation, fill their menstrual knowledge gap, and clears up misconceptions they may have about menstruation and menstrual blood [31].

Furthermore, educating men and boys on menstruation may create an environment to engage in a more open conversation about menstruation and enhance support from men and boys during a woman’s or a girls period of menstruation [31]. Consistent with the findings of a study in India [34], most of the boys in this study obtained information about menstruation from schoolteachers. However, there are concerns that schoolteachers may not provide detailed information on menstruation in schools, particularly in mixed-gender classes, because of the taboos and restrictions associated with menstruation in low and middle-income settings [3]. This may further explain why the boys in this study did not have an in-depth understanding of menstruation and the biological processes involved.

Comfort, safety, and cost were the major factors that influenced the choice of menstrual absorbent materials among the girls in this study. Even though we found that sanitary pads were sold in shops in the study area, 46% of the study participants had not bought sanitary products from the shops two months before the study. Cost of sanitary products was the most common reason cited by the majority (75.7%) of the respondents for their inability to purchase the product which is in line with the findings of a similar study in Uganda [21]. Additionally, the schoolteachers indicated that the schools do not provide sanitary pads to students. This situation may compel the girls to use non-absorbent and uncomfortable materials to absorb menstrual blood, which may affect their school attendance or participation in physical activities when they are menstruating, usually due to fear of leakage and the ensuing embarrassment and teasing from their colleagues [37]. For instance, in Zambia, girls who used non-absorbent materials complained of friction burns on their inner thighs [37].

Adequate and suitable sanitary materials could empower women and girls to feel more confident and comfortable with their bodies [31]. Some of the boys wrongly thought menstrual absorbent materials are used to suppress unpleasant odour from the menstrual blood. This underscores the need to involve boys in menstrual hygiene education as the inclusion of boys will provide them with accurate knowledge about menstruation and dispel misconceptions and myths they may have about menstruation and people who menstruate.

Several studies have shown that negative cultural practices and taboos around menstruation have harmful effects on the lives of women and girls and strengthen gender inequities and exclusion [2, 9, 13]. In the present study, we found that girls were forbidden from participating in religious activities during the period of menstruation. Adolescent boys confirmed this practice. This finding is consistent with a study in Nepal where the common restriction among the student respondents was abstinence from religious activities [9]. In the current study area, open discussions about menstruation and its management were not encouraged and girls were considered unclean and impure during the period of menstruation. Likewise, women and girls in the Gambia expressed difficulties, embarrassment, and shame with discussing menstruation, and as a result, two-thirds of the girls in the study felt unprepared for menstruation and menstrual hygiene at menarche [8]. This highlights the point that the taboos, secrecy, and the embarrassment associated with discussing menstruation prevent adolescents from seeking accurate information from parents and teachers on appropriate menstrual hygiene management practices and may cause the young girls to grow up with limited information about menstruation and menstrual management [4, 8]. The adolescent boys revealed that it is taboo for menstruating women and girls to cook ‘*Wasawasa*’, a local dish prepared from yam and common to the people of northern Ghana. It is believed that the dish does not like uncleanness and since menstruating women and girls are considered unclean and impure, they are to take a spiritual bath to clean their body after menstruating before they are permitted to prepare the dish.

Essential to good menstrual hygiene management in schools is the provision of safe, private, and clean water, sanitation, and hygiene (WASH) facilities for changing menstrual materials, washing and drying materials, discrete disposal options, and soap to maintain personal hygiene [6, 10, 38]. All the Junior High Schools in the current study area had separate toilet blocks for boys and girls, and the toilets and wash facilities had doors but most of the doors lacked locks. Teachers and adolescent boys confirmed this observation. In addition, only two of the five schools had clean toilets which is similar to what was found in schools in Nepal where toilets were often not clean or private [3]. Consistent with the findings of studies from Zambia and Mali [37, 39], none of the schools we surveyed had a mirror, regular supply of water and soap in their toilet facilities.

Most of the schools in the current study area had standpipes outside the toilets and students were expected to carry water to the toilet for use. This finding is congruent with an earlier study in Ghana where water sources in schools were not connected to toilets facilities and girls had to fetch water into the toilets to wash or change when necessary [14]. Further, only two of the schools had functioning hand-washing facilities (veronica buckets). The handwashing facilities were positioned outside the toilets.

The lack of adequate and suitable sanitation and hygiene facilities could lead to girls experiencing shame, embarrassment, discomfort, and may cause girls to miss school during menstruation [4, 6, 38]. Findings from earlier studies revealed that girls from schools with lack of water and poor privacy in toilets are not comfortable attending school during the period of menstruation [19, 32]. Even when girls attend school during their periods they are usually compelled by the inadequate sanitary facilities to leave school early to change their used pads at home [3].

## Conclusion

The findings of the study highlight the point that there are still misconceptions, taboos, and myths about menstruation and the people who menstruate. In addition, schools are not doing enough to provide a suitable and supportive environment for adolescent girls during the period of menstruation. To address the misconceptions about menstruation, girls should be educated on menstruation and proper menstrual hygiene practices through the standard school curriculum starting from primary school. Schoolteachers should be prepared with accurate information on menstruation and supported with the needed resources to deliver appropriate menstruation education in schools. Menstrual education in schools should include boys for them to appreciate the process of menstruation and learn how to support girls and women during the period of menstruation. Besides education, schools should be able to support girls with menstrual hygiene products especially during menstrual emergencies and provide appropriate and functional water, sanitation, and hygiene (WASH) facilities to ensure hygienic management of menstruation in schools. It is also essential to increase the awareness of mothers about menstruation and its hygienic management. Therefore, menstrual education should be incorporated into routine health promotion and health education programmes in the communities to provide accurate and practical information to mothers on menstruation.

## Supporting information

S1 Questionnaire (PDF)

## List of abbreviations

FGD: Focus group discussion
KII: key informant interviews
MHM: menstrual hygiene management
JHS: Junior High School
WASH: Water, sanitation, and hygiene
CAMFED: Campaign for Female Education
NGO: Non-governmental organisation

## References

[1] Sommer M, Sahin M. Overcoming the Taboo: Advancing the Global Agenda for Menstrual Hygiene Management for Schoolgirls. Am J Public Health. 2013;103(9):1556–9. 10.2105/AJPH.2013.301374 23865645 PMC3780686

[2] House S, Mahon T, Cavill S. Menstrual hygiene matters: A resource for improving menstrual hygiene around the world. First Edit. London, UK: WaterAid; 2012 1–354 p.

[3] Morrison J, Basnet M, Bhatta A, Khimbanjar S, Baral S. ANALYSIS OF MENSTRUAL HYGIENE PRACTICES IN NEPAL: The Role of WASH in Schools Programme for Girls Education 2016. Nepal; 2018.

[4] UNICEF. sharing simple facts: Useful information about menstrual health and hygiene. New Delhi, India: UNICEF India; 2008.

[5] Ten VTA. Menstrual Hygiene: A Neglected Condition for the Achievement of Several Millennium Development Goals. Ten VTA, editor. Brussels, Belgium: Europe External Policy Advisors; 2007. 1–24 p.

[6] Alberda H. Menstrual Health: Training Manual. The Netherlands; 2018.

[7] WSSCC and UN Women. MENSTRUAL HYGIENE MANAGEMENT: BEHAVIOUR AND PRACTICES IN THE LOUGA REGION, SENEGAL. Geneva, Switzerland; 2014.

[8] Shah V, Nabwera HM, Sosseh F, Jallow Y, Comma E, Keita O, et al A rite of passage: a mixed methodology study about knowledge, perceptions and practices of menstrual hygiene management in rural Gambia. BMC Public Health. 2019;19(277):1–15. 30845945 10.1186/s12889-019-6599-2 PMC6407285

[9] WaterAid. Is menstrual hygiene and management an issue for adolescent school girls? WaterAid/Anita Pradhan A comparative study of four schools in different settings of Nepal [Internet]. Kupondole, Lalitpur, Nepal; 2009. Available from: www.wateraid.org/nepal

[10] Swedish International Development Cooperation Agency [Sida]. Menstrual Hygiene Management. In: Health: Both a prerequisite and an outcome of sustainable development. stockholm, sweden: SWEDISH INTERNATIONAL DEVELOPMENT COOPERATION AGENCY; 2016. p. 1–3.

[11] Um L, Yusuf NW, Musa AB. Menstruation and Menstrual Hygiene amongst Adolescent School Girls in Kano, Northwestern Nigeria. Afr J Reprod Health. 2010;44(3):201–8. 21495614

[12] Thakre subhash b., Thakre sushama s., Reddy M, Rathi N, Pathak K, Ughade S. Menstrual Hygiene: Knowledge and Practice among Adolescent School Girls of Saoner, Nagpur District. J Clin Diagnostic Res [Internet]. 2011;5(5):1027–33. Available from: http://jcdr.net/back_issues.asp?issn=0973-709x&year=2011&month=October&volume=5&issue=5&%0Apage=1027-1033&id=1522=0973-709x&year=2011&month=October&volume=5&issue=5&%0Apage=1027-1033&id=1522

[13] Kennedy E (Burnet I), Suriastini W (SurveyMETER), Macintyre A (WaterAid A), Huggett C (WaterAid A), Wheen R (WaterAid A), Faiqoh F (Aliansi RI), et al. Menstrual Hygiene Management in Indonesia: Understanding practices, determinants, and impacts among adolescent school girls [Internet]. Indonesia; 2015. Available from: https://www.burnet.edu.au/system/asset/file/2034/2015_Menstrual_hygiene_management_Indonesia_FINAL_REPORT_February_2015_low_res.pdf.

[14] Abanyie SK, Anang RC, Ampadu B. Menstrual Health Management in Some Selected Basic Schools in Ghana. In: ENSURING AVAILABILITY AND SUSTAINABLE MANAGEMENT OF WATER AND SANITATION FOR ALL. Kumasi, Ghana: wedc.lboro.ac.uk/resources/conference/39/Abanyie-2389.pdf; 2016. p. 1–6.

[15] Sapkota D, Sharma D, Budhathoki S, Khanal V, Pokharel H. school going adolescents of rural Nepal. Orig Artic J Kathmandu Med Coll. 2013;2(3):2–8.

[16] Kumar A, Srivastava K. Cultural and Social Practices Regarding Menstruation among Adolescent Girls Cultural and Social Practices Regarding Menstruation among Adolescent Girls. Soc Work Public Health. 2011;26(6):594–604. 10.1080/19371918.2010.525144 21932979

[17] Sommer M, Ackatia-armah N, Connolly S, Smiles D. A comparison of the menstruation and education experiences of girls in Tanzania, Ghana, Cambodia and Ethiopia. J Comp Int Educ. 2015;45(4):589–609.

[18] UNESCO. Puberty Education & Menstrual Hygiene Management. Booklet 9. Paris, France: United Nations Educational, Scientific and Cultural Organization; 2014.

[19] Mohammed S, Larsen-Reindorf RE, Awal I. Menstrual Hygiene Management and School Absenteeism among Adolescents in Ghana: Results from a School-Based Cross-Sectional Study in a Rural Community. Int J Reprod Med. 2020;2020:1–9. 10.1155/2020/6872491 PMC7204135 32411782

[20] Tegegne TK, Sisay MM. Menstrual hygiene management and school absenteeism among female adolescent students in Northeast Ethiopia. BMC Public Health [Internet]. 2014;1–14. Available from: http://www.biomedcentral.com/1471-2458/14/1118 10.1186/1471-2458-14-1 25355406 PMC4232635

[21] Boosey R, Prestwich G, Deave T. Menstrual hygiene management amongst schoolgirls in the Rukungiri district of Uganda and the impact on their education: a cross-sectional study. Pan African Med Journa. 2014;253(19):1–13. 10.11604/pamj.2014.19.253.5313 PMC4382073 25852796

[22] Coast E, Lattof SR, Strong J. Puberty and menstruation knowledge among young adolescents in low- and middle-income countries: a scoping review. Int J Public Health. 2019;64(2):293–304. 10.1007/s00038-019-01209-0 30740629 PMC6439145

[23] Chang Y, Hayter M, Lin M. Pubescent male students ‘ attitudes towards menstruation in Taiwan: implications for reproductive health education and school nursing practice. J Clin Nurs. 2011;21:513–21. 10.1111/j.1365-2702.2011.03700.x 21457380

[24] Ghana Statistical Service (GSS). 2010 Population & Housing Census. District Analytical Report: Kumbungu District. Accra, Ghana; 2014.

[25] Kasiulevičius V, Šapoka V, Filipavičiūtė R. Sample size calculation in epidemiological studies. Gerontologija. 2006;7(4):225–31.

[26] Ameade EPK, Garti HA. Relationship between Female University Students’ Knowledge on Menstruation and Their Menstrual Hygiene Practices: A Study in Tamale, Ghana. Adv Prev Med. 2016;2016:1–10. 10.1155/2016/1056235 PMC4976185 27525125

[27] UNICEF. Supporting the Rights of Girls and Women through Menstrual Hygiene Management (MHM) in the East Asia and Pacific Region Good Practice Guidance Note [Internet]. Bangkok, Thailand: UNICEF East Asia and Pacific Regional Office (EAPRO); 2016. 1–36 p. Available from: www.unicef.org/eapro

[28] Upashe SP, Tekelab T, Mekonnen J. Assessment of knowledge and practice of menstrual hygiene among high school girls in Western Ethiopia. BMC Womens Health. 2015;15(84):1–8. 26466992 10.1186/s12905-015-0245-7 PMC4606849

[29] UNICEF. Water, Sanitation and Hygiene (WASH) in Schools. Mooijman A, editor. NewYork, USA: United Nations Children’s Fund (UNICEF); 2012. 3–4 p.

[30] Crofts T. Menstruation hygiene management for schoolgirls in low - income countries [Internet]. Fact Sheet. Leicestershire, UK: Water, Engineering and Development Centre; 2012. Available from: http://wedc.lboro.ac.uk/resources/factsheets/FS007_MHM_A4_Pages.pdf.

[31] Mathiaud C. Menstrual Hygiene Matters: Menstrual Hygiene Insecurity (MHI) and the need for a global recognition of adequate Menstrual Hygiene Management (MHM) as a fundamental right for all women and girls. France; 2014.

[32] Kgware M. Menstruation and menstrual hygiene management in selected kwazulu-natal schools. Martin C, editor. South Africa: Oxfam; 2016.

[33] Chandra-mouli V, Patel SV. Mapping the knowledge and understanding of menarche, menstrual hygiene and menstrual health among adolescent girls in low- and middle-income countries. Reprod Health. 2017;14(30):1–16. 28249610 10.1186/s12978-017-0293-6 PMC5333382

[34] Mason L, Sivakami M, Thakur H, Kakade N, Beauman A, Alexander KT, et al ‘ We do not know ‘: a qualitative study exploring boys perceptions of menstruation in India. Reprod Health. 2017;14(174):1–9. 29216895 10.1186/s12978-017-0435-x PMC5721687

[35] Nanda G, Lupele J, Tharaldson J. Menstrual Hygiene Management among Schoolgirls in Eastern Province of Zambia. USAID/WASHplus Project; 2016.

[36] Johnson L, Calderón T, Hilari C, Long J, Vivas C. Menstrual Hygiene Management Impacts Girls ‘ School Experience in the Bolivian Amazon Photo credits. La Paz Bolivia; 2016.

[37] Chinyama J, Chipungu J, Rudd C, Mwale M, Verstraete L, Sikamo C, et al Menstrual hygiene management in rural schools of Zambia: a descriptive study of knowledge, experiences and challenges faced by schoolgirls. BMC Public Health. 2019;19(16):1–10. 30611223 10.1186/s12889-018-6360-2 PMC6321718

[38] The World Bank. Menstrual Hygiene Management Enables Women and Girls to Reach Their Full Potential. 2018.

[39] Trinies V, Caruso BA, Freeman MC. Uncovering the challenges to menstrual hygiene management in schools in Mali. Waterlines. 2015;34(1):32–40.

