# Supplementary material for: Menstrual knowledge, sociocultural restrictions, and barriers to menstrual hygiene management in Ghana: Evidence from a multi-method survey among adolescent schoolgirls and schoolboys
Source: PLoS One. 2020 Oct 22;15(10):e0241106. doi: 10.1371/journal.pone.0241106 (PMC7580927; doi:10.1371/journal.pone.0241106)
Supplement: S1 Questionnaire — (PDF) [file pone.0241106.s001.pdf]

APPENDIX 1

Name of your School ____<br><br>Questionnaire Identification Number___________________________

No	Socio-Demographic Variables<br>Instruction: circle the responses or write the appropriate answer in the space provided	
Q1	What was your age at your last birthday?	______________Complete years
Q2	Religion	1. Christian [ ]<br>2. Muslim [ ]<br>3. Traditionalist [ ]<br>4. Other (specify)___________
Q3	At what age did you have your first menstrual period	_______________Years
Q4	Does your family have a TV or/and radio?	1. Yes<br>2. No
Q5	What is the occupation of your father/male guardian?	1. Tailor<br>2. Trader<br>3. Farmer<br>4. Daily labourer<br>5. Formal sector employee<br>6. Unemployed<br>7. Other (specify) _____________
Q6	What is the occupation of your mother/female guardian?	1. Tailor<br>2. Trader<br>3. Farmer<br>4. Daily labourer<br>5. Formal sector employee<br>6. Unemployed<br>7. Other (specify) _____________
Q7	What is the education level of your father/male guardian?	1. No education [ ]<br>2. Basic [ ]<br>3. Secondary [ ]<br>4. Tertiary [ ]
Q8	What is the education level of your mother/female guardian?	1. No education [ ]<br>2. Basic [ ]<br>3. Secondary [ ]<br>4. Tertiary [ ]

No	Menstrual Knowledge<br>**Instruction**: circle the responses or write the appropriate answer in the space provided	
Q9	Menstruation is a…	1. Normal healthy process in girls and women<br>2. Not a normal healthy process in girls and women<br>3. Other (Specify) _______________
Q10	Menstrual blood comes from which body part?	1. Abdomen<br>2. Bladder<br>3. Vagina<br>4. Womb (uterus)<br>5. Other (Specify)_________________
Q11	At what age, do you think most girls usually get their first period?	a. ________________ years<br>b. Don't know
Q12	How many days should a girl's menstrual bleeding last?	a. _____________ Days<br>b. Don't know
Q13	What is the length of the menstrual cycle?	a. _____________ Days<br>b. Don't know
Q14	Did anyone tell you about menstruation before you started menstruating?	1. Yes<br>2. No
Q15	If your response to No.15 is yes, from whom did you first learn about menstruation? (More than one answer is possible)	1. Mother<br>2. Teacher<br>3. Friends<br>4. Books<br>5. Media (TV, Radio)<br>6. Relatives (Aunt, grandmother, sister etc)<br>7. Others (Specify)_______________
Q16	What kinds of foods should be avoided during periods?	1. Sugary foods<br>2. Oily foods<br>3. Milk products<br>4. No food restrictions<br>5. Meats<br>6. Eggs<br>7. Snails<br>5. Others (specify)___________________

Menstrual hygiene management materials<br>Instruction: circle the responses or write the appropriate answer in the space provided		
Q17	What absorbent materials do you frequently use during menstruation? (More than one answer is possible)	1. Reusable cloth pad<br>2. Disposable sanitary pad<br>3. Disposable rag or piece of Cloth<br>4. Cotton wool<br>5. Underwear/pant<br>6. Toilet paper<br>7. Paper (newspaper, pages from books)<br>8. Other (specify)_________________
Q18	What influences your choice of menstrual absorbent materials?	1. Comfort<br>2. Safety<br>3. Cost<br>4. Availability<br>5. Ease of disposal<br>6. Ease of re-use<br>7. Other (specify)_______________
Q19	Are sanitary pads available for sale in the shops in your town?	1. Yes<br>2. No<br>3. Don't know
Q20	Have you bought disposable sanitary pads from a shop in the last two months?	1. Yes<br>2. No
Q21	If no, why have you not bought some?	1. I still have some pads<br>2. I don't have money<br>3. My parents buy for me<br>4. It is not yet time to buy for next period<br>5. I am embarrassed to purchase pads<br>6. Others (Specify)_________________

Cultural and Religious Restrictions on Menstruation		Yes	No	I don't know
Q22	It is prohibited to openly discuss menstruation and its management in my culture			
Q23	Menstruating girls are confine to a room or a separate menstruation hut to avoid interaction with men			
Q24	Menstruating girls are prohibited from performing house chores such as cooking, fetching water, sweeping etc			
Q25	Menstruating girls are forbidden from religious activities (praying, visits to mosque or church, forbidden from touching religious books etc.)			
Q26	Menstruating girls are considered unclean and impure			
